# Anatomical Transcriptome Atlas of the Male Mouse Reproductive System During Aging

**DOI:** 10.3389/fcell.2021.782824

**Published:** 2022-02-08

**Authors:** Yanping Huang, Xiangping Li, Xiangzhou Sun, Jiahui Yao, Fengxin Gao, Zhenqing Wang, Jiaying Hu, Zhu Wang, Bin Ouyang, Xiangan Tu, Xuenong Zou, Wei Liu, Mujun Lu, Chunhua Deng, Qiyun Yang, Yun Xie

**Affiliations:** ^1^ Department of Urology and Andrology, Renji Hospital, School of Medicine, Shanghai Institute of Andrology, Shanghai Jiao Tong University, Shanghai, China; ^2^ Department of Urology and Andrology, The First Affiliated Hospital, Sun Yat-sen University, Guangzhou, China; ^3^ Guangzhou Epibiotek Co., Ltd., Guangzhou, China; ^4^ Department of Ultrasonics, Institute of Diagnostic and Interventional Ultrasound, The First Affiliated Hospital, Sun Yat-Sen University, Guangzhou, China; ^5^ Department of Andrology, Guangzhou First People’s Hospital, School of Medicine, South China University of Technology, Guangzhou, China; ^6^ Guangdong Provincial Key Laboratory of Orthopedics and Traumatology, Department of Spinal Surgery, The First Affiliated Hospital, Sun Yat-sen University, Guangzhou, China

**Keywords:** male reproductive system, reproductive aging, transcriptome, testis, epididymis, PLA2G2D

## Abstract

The elderly males undergo degenerative fertility and testicular endocrine function that jeopardize the reproductive health and well-being. However, the mechanisms underlying reproductive aging are unclear. Here, we tried to address this by investigating the phenotypes and transcriptomes of seven regions of the male mouse reproductive tract: the testis, efferent ductules, initial segment, caput, corpus and cauda epididymidis, and vas deferens, in adult (3 months) and aged (21 months) mice. Quantitative PCR, immunohistochemistry, immunofluorescent staining, and enzyme-linked immunosorbent assay were performed for the analysis of gene expression in mice, human tissues, and semen samples. Aged male mice showed both systematic and reproductive changes, and remarkable histological changes were detected in the testis and proximal epididymis. Transcriptomes of the male reproductive tract were mapped, and a series of region-specific genes were identified and validated in mouse and/or human tissues, including Protamine 1 (*Prm2*), ADAM metallopeptidase domain 28 (*Adam28*), Ribonuclease A family member 13 (*Rnase13*), WAP four-disulfide core domain 13 (*Wfdc13*), and *Wfdc9*. Meanwhile, age-related transcriptome changes of different regions of the male reproductive tract were characterized. Notably, increased immune response was functionally related to the male reproductive aging, especially the T cell activation. An immune response-associated factor, phospholipase A2 group IID (*Pla2g2d*), was identified as a potential biomarker for reproductive aging in mice. And the PLA2G2D level in human seminal plasma surged at approximately 35 years of age. Furthermore, we highlighted Protein tyrosine phosphatase receptor type C (*Ptprc*), Lymphocyte protein tyrosine kinase (*Lck*), Microtubule associated protein tau (*Mapt*), and Interferon induced protein with tetratricopeptide repeats 3 (*Ifit3*) as critical molecules in the aging of initial segment, caput, caput, and cauda epididymidis, respectively. This study provides an RNA-seq resource for the male reproductive system during aging in mice, and is expected to improve our understanding of male reproductive aging and infertility.

## Introduction

Aging is a progressive process involving gradual declines in the functions of multiple organs and cells. A delay in the age of paternity due to social factors has resulted in aging-related fertility concerns (Sharma et al., 2015). In elderly men, fertility and testicular endocrine function decline. Advanced age is negatively correlated with sperm concentration, motility, normal morphological changes, serum testosterone concentration, and reproductive outcomes (Sharma et al., 2015; Gunes et al., 2016). The sperm of elderly men are more likely to bear genetic and epigenetic defects, leading to an elevated risk of pregnancy loss and birth defects in offspring (Yoon et al., 2009; Kong et al., 2012; Paoli et al., 2019; Potabattula et al., 2020). Moreover, due to steroidogenic dysfunction in the testis (or late-onset hypogonadism), there is also an increased incidence of a variety of diseases in elderly men, including type 2 diabetes, osteoporosis, and Alzheimer’s disease (Henderson and Hogervorst, 2004; Basaria, 2014). However, the exact mechanisms underlying male reproductive aging remain largely unclear.

The testis, efferent ductules, epididymidis, and vas deferens (also known as the ductus deferens) constitute the major parts of the male reproductive tract and are responsible for testosterone production, spermatogenesis, and sperm maturation, storage, and discharge (Nixon et al., 2020). Following spermatogenesis in the testis, the efferent ductules transport immotile sperm from the rete testis to the proximal epididymidis by generating luminal turbulence with the whip-like beating of motile cilia (Yuan et al., 2019). Subsequently, sperm are capable of forward motility and fertilization when passing through the epididymis; they are stored mainly in the cauda epididymidis and discharged through the vas deferens. In rodents, the epididymis can be anatomically divided into 10 and 19 segments in mouse and rat, respectively (Turner et al., 2003; Johnston et al., 2005; Jelinsky et al., 2007; Nakata and Iseki, 2019), with distinct morphologies, distributions of cell types, and gene expression profiles (Sipilä and Björkgren, 2016; Rinaldi et al., 2020). Therefore, a comprehensive understanding of the transcriptome profile of the male reproductive tract is crucial. Even so, few of RNA-seq data has been published to systematically map the male reproductive system during aging, including region-specific genes and age-related genes. With advances in the single-cell RNA sequencing (scRNA-seq) technique, it is currently possible to reveal the transcriptome changes at a single-cell resolution within highly heterogeneous tissues such as female gonad (ovary) (Wang et al., 2020). Alternatively, considering the complex anatomical structure of male reproductive tract, the high cost, and a bulk of data output of scRNA-seq, it is still necessary to capture a baseline data of transcriptomics.

To investigate the age-related changes in the male reproductive tract, we systematically studied the phenotypic, histological, and transcriptomic characteristics in a murine model of natural aging: the testis, efferent ductules, initial segment, caput, corpus, and cauda epididymidis, and vas deferens. We identified the transcriptome changes of male reproductive tract in a region-dependent manner. The analyses gave the first general landscape of the male reproductive aging which may help us narrow down the anatomical regions of interest for further study. Moreover, we explored the clinical and translational significance of these data sets within humans, and screened potential region-specific or age-related biomarkers in human seminal plasma.

## Materials and Methods

### Animals and Biological Samples

A total of 30 C57BL/6 male mice were purchased from the experimental animal center of Sun Yat-sen University (Guangzhou, China) and Xiamen University (Xiamen, China), and maintained in specific pathogen-free (SPF) environment (12:12 h light/dark cycle) with free access to food and water. Human semen samples were collected from 145 consecutive male outpatients (aged 30–50 years) for fertility examination with normal semen parameters that meet the standard of the fifth edition of the World Health Organization manual of semen analysis (Cooper, 2010). All patients were abstinent for 2–7 days before examination. The semen samples were obtained by masturbation and allowed to liquefy at 37°C for 20 min. Seminal plasma was obtained after centrifugation (3,000 × *g*, 15 min). Human testis tissues for immunofluorescent staining analysis were collected from 3 obstructive azoospermia patients by diagnostic testicular biopsy.

### Muscle Strength and Bone Mineral Density Measurement

Before euthanasia, the muscle strength of limbs was assessed by a wire screen holding test described previously (Carlson et al., 2010). The muscle strength was evaluated by the physical impulse (Fdt) (N·s) = body mass (g) × 0.00980665 N/g × holding time(s). After euthanasia with an intraperitoneal injection of excess pentobarbital sodium, the femurs and tibias were harvest. And the Bone mineral density was measured using a Siemens Inveon Micro-CT scanner (Siemens Medical Solutions United States Inc, Malvern, PA, United States).

### Sperm Count and Motility Measurement

The cauda epididymidis (2 mm in length) was minced in 1 ml Biggers, Whitten, and Whittingham medium (BWW) containing 0.5 ml 4% bovine serum albumin (BSA) prewarmed to 37°C, then cut into small pieces and incubated for 10 min at 37°C to release the sperm. Sperm concentration and motility were measured with a computer aided sperm analysis (CASA) system (Hamilton Thorne, Beverly, MA, United States). The average of 5 visual fields was calculated.

### Testosterone Concentration Assay

Blood samples were collected from mice after euthanasia. Serum was obtained after centrifugation (3,000 × g, 15 min, 4°C), and stored at −70°C until assayed. The testosterone concentrations were measured by electrochemiluminescence immunoassay using Elecsys Testosterone II (05200067190, Roche, Basel, Switzerland) according to the manufacturer’s instructions.

### RNA-Seq Analysis

Four mice each of 3 months old and 21 months old were used for RNA sequencing analysis. A pair of bilateral testes, efferent ductules, epididymides and vas deferens of the same mouse was dissected into a 10-cm plate containing 10 ml Phosphate Buffered Saline (PBS). After the removal of any fat and excessive connective tissue, seven regions of the male reproductive tract: testis, efferent ductules, initial segment, caput epididymidis, corpus epididymidis, cauda epididymidis, and vas deferens, were isolated under a dissection microscope as previously described (Nakata and Iseki, 2019). Total RNA was extracted using a RNeasy mini kit (Qiagen, Hilden, Germany) according to the manufacturer’s protocol. The library was prepared using Illumina TruSeq Stranded Total RNA Library Prep Kit (Illumina Inc., San Diego, CA, United States), and paired-end 150 bp RNA-seq was performed on Illumina NovaSeq (6,000 (Illumina Inc.). Reads were aligned with Hisat2 to the mouse genome (Ensemble Mouse GRCm38). Differential gene expression was assessed using DEseq2 R package. High-throughput sequencing of exosomal mRNA in seminal plasma was conducted according to the method reported in our previous study (Xie et al., 2020).

### Quantitative PCR

Total RNA was extracted from tissues or semen plasma using a RNAiso Plus (9,109, Takara, Japan) according to the manufacturer’s protocol. Total RNA (2 µg) was used as a template to synthesize cDNA with PrimeScrip RT Master Mix Kit (RR036A; Takara, Japan) at 37°C for 15 min and 85°C for 5 s. The qPCR was performed using the TB Green Premix Ex Taq II (RR820A, Takara, Japan) according to the manufacturer’s instructions. The qPCR program using the LightCycler 480 II real-time PCR machine (Roche, Basel, Switzerland) was then carried out as follows: initial denaturation at 95°C for 30 s and 80 cycles of 95°C for 5 s and 60°C for 20 s; with a final step melting curve of 95°C for 5 s; 60°C for 1 min and 95°C for 5 s. All the primers used are listed in Supplementary Table S1 (Sangon Biotech, Shanghai, China).

### Histological Analyses

The mouse testis, efferent ductules, initial segment, caput, corpus and cauda epididymidis, and vas deferens were fixed in 4% paraformaldehyde. After steps of dehydration, paraffin-embedding, slices at 5-μm thickness were used for hematoxylin and eosin (H&E) staining. β-galactosidase staining in frozen mouse testis and epididymis tissues was performed using a senescence β-galactosidase staining kit (G1073-100T, Servicebio, Wuhan, China) according to the manufacturer’s instructions.

Paraffin slides of the mouse testis, epididymidis, and human testis tissues were deparaffinized in xylene and then rehydrated in graded alcohol solutions. The endogenous peroxidase activity was inhibited by incubating in 3% H_2_O_2_, and the antigenicity was recovered with 1 × Tris-EDTA buffer (pH 9.0, G1203, Servicebio, Wuhan, China). The samples were permeabilized with 0.3% Triton-X100 for 10 min, then incubated with 5% Bovine Serum Albumin for 30 min. For immunohistochemistry staining in mouse testis, the primary antibody included anti-Pla2g2d (1:100, PA5-93147, Invitrogen, United States) was used. PBS buffer was used as negative control. After washed with PBS then incubated with HRP Conjugated Goat Anti-Rabbit IgG (1:100, CW0103, CWBIO, Beijing, China) at room temperature for 30 min, the sections were counterstained with Hematoxylin Staining Solution (ZLI-9610, ZSGB-BIO, Beijing, China). For immunofluorescent staining in mouse epididymis and human testis, the primary antibody included anti-Pla2g2d (1:100, PA5-93147, Invitrogen, United States) and anti-PRM2 (1:100, 14500-1-AP, Proteintech Group, Inc, China) were used. After washed with PBS then incubated with CY3 Conjugated AffiniPure Goat Anti-rabbit IgG (1:100, BA1032, Boster Biological Technology co.ltd, Wuhan, China) at room temperature for 30 min, the sections were counterstained with DAPI (10 μg/ml, C1002, Beyotime Biotechnology, Shanghai, China). Immunofluorescent staining in human sperm was conducted according to previous study (Liu et al., 2015). Images were captured with a Leica inverted fluorescence microscope (Leica, Wetzlar, Germany) or an Olympus inverted microscope (Olympus, Tokyo, Japan).

### Enzyme-Linked Immunosorbent Assay

Human seminal plasma was obtained after centrifugation (3,000 × g, 15 min, 4°C). The supernatant was centrifuged at 12 000 × g for 30 min at 4°C to remove cellular debris. The PLA2G2D levels of 40 samples from different ages (30–50 years) were determined using an ELISA kit according to the manufacturer’s instructions (LS-F4416, LifeSpan BioSciences Inc., Seattle, WA, United States).

### Bioinformatics Analysis

Gene Ontology (GO) enrichment analysis was performed using the Gene Ontology resource database (http://geneontology.org/) and visualized using R language packages. The GO terms with a FDR-adjusted P-value less than 0.01 were thought significant enriched. The PPI network was constructed using STRING database (https://www.string-db.org/) and visualized by Cytoscape software (https://cytoscape.org/). Upset plot and GO Correlation Network plot were performed using the OmicStudio tools at http://www.omicstudio.cn/.

### Statistical Analysis

SPSS 26.0 statistical software (IBM Corp., NY, United States) was used for statistical analysis. All data were presented as the mean ± standard deviation (SD) obtained from at least four independent experiments. Comparisons between groups were performed using Student’s t-test, and *p* < 0.05 was considered statistically significant.

## Results

### Old Male Mice Demonstrate Systemic and Reproductive Aging

**FIGURE 1:**
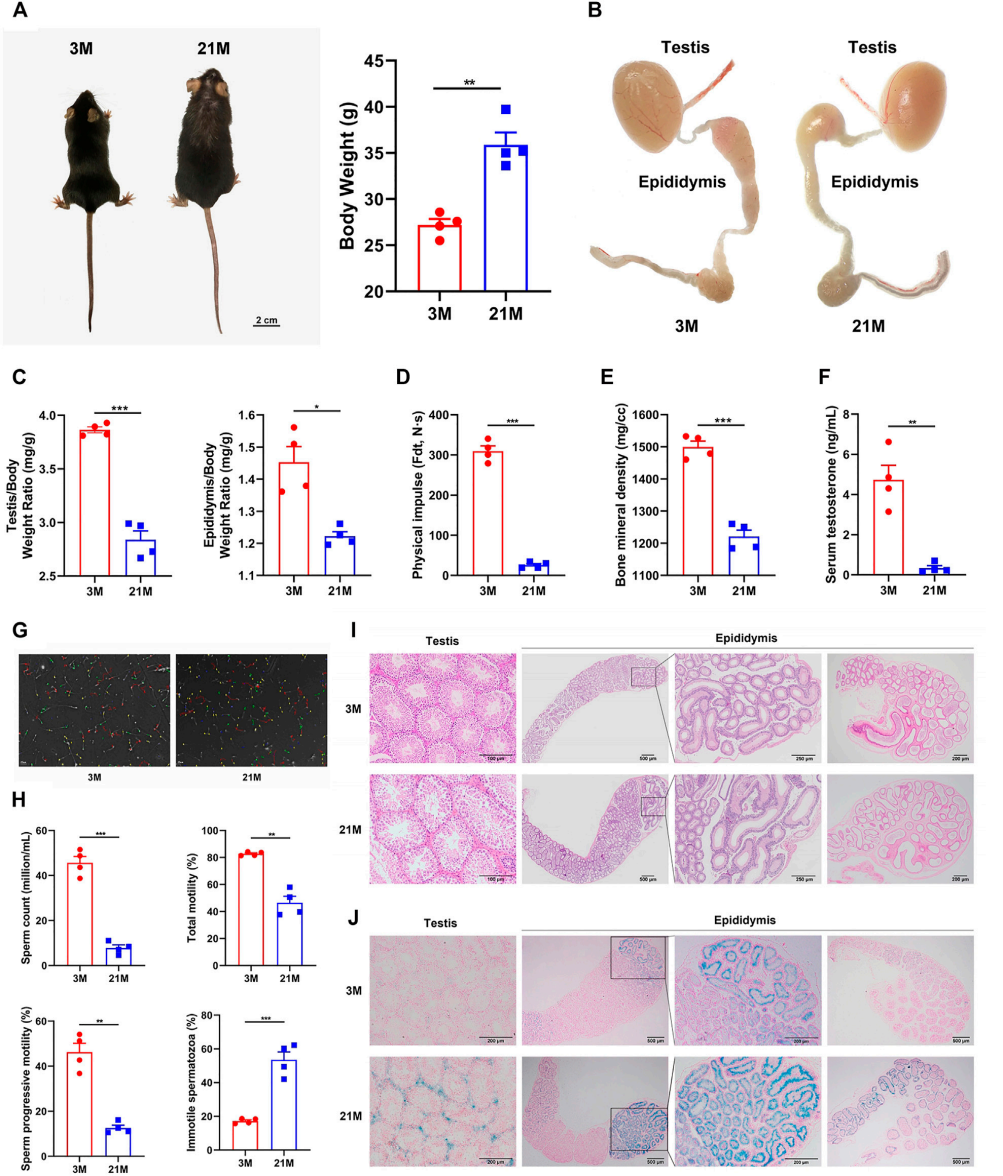
Phenotypic and histological analyses of reproductive and systemic aging in male mice. **(A)** Physical appearance and body weight of mice of 3-months and 21-month. **(B)** Male reproductive organs in mice of different ages. **(C)** Organ indices of the testis and epididymis at different ages. **(D)** Muscle strength of limbs calculated by physical impulse, **(E)** bone mineral density of the femur, **(F)** serum testosterone concentration, and **(G–H)** epididymal sperm concentration and motility between young and old mice. **(I)** Representative hematoxylin and eosin staining of testis and epididymis. **(J)** Representative β-Galactosidase staining of the testis and epididymis. Strong positive staining was detected in the testicular interstitium (lower left panels), the initial segment of epididymis, and a small area of the caput epididymidis connected to the initial segment (lower right panels) in aged mice. Weak positive staining was detected in the epithelium of the corpus epididymidis (lower right panels) and was barely observed in the cauda epididymidis (lower right panels, the far-right picture). All experiments were performed in at least 4 independent replicates. *p < 0.05, **p < 0.01, ***p < 0.001; 3 M: 3-months; 21 M: 21-months.

To determine whether 21-month-old male mice are a suitable model for the natural reproductive aging, we comprehensively studied phenotypes associated with reproductive aging as well as systemic aging. The 21-month-old male mice exhibited physical features of increased body weight and loss of hair and luster (Figure 1A). Both testis and epididymis indices (organ weights relative to the mouse body weight) were significantly decreased (*p* < 0.05) (Figures 1B,C). However, no evident morphological differences were detected between the young and elderly mice, except for a decrease in plumpness with age (Figure 1B). Noticeably, the initial segment had the richest blood supply across the epididymis at both ages (Figure Figure1B). Furthermore, the limb muscle strength, a simple indicator of systemic aging and the bone mineral density of the femur decreased significantly with age (*p* < 0.05) (Figures 1D,E). The serum testosterone concentration, epididymal sperm concentration, and motility decreased remarkably in the elderly group (*p* < 0.05) (Figures 1F–H). Collectively, the 21-month-old male mice showed characteristics of both systemic and reproductive aging.

### Dramatic Histological Changes in the Testis and Proximal Epididymis with Age

Previous analyses paid little attention to the histological characteristics of male reproductive tract aging, and it is unclear which region responsible for reproductive function arises age-related changes. To investigate the biological basis for the reproductive aging in mice, we further performed histological analyses on the testis and epididymis by Hematoxylin and eosin (HE) staining and β-Galactosidase (β-Gal) staining. The testes of elderly mice had dilated and empty lumens of the seminiferous tubules, as well as disordered and thinner seminiferous epithelium (Figure 1I, left panel). In the epididymis, dilatation of the lumen was also observed with age. Notably, the arrangement of ducts in the initial segment and caput epididymidis was compact in the young group; in contrast, it was much looser in the elderly group (Figure 1I, right panel). Moreover, β-galactosidase (β-Gal) in the testis and epididymis showed strong positive staining in the testicular interstitium, the initial segment of the epididymis, and a small area of caput epididymidis connected to the initial segment in aged mice (Figure 1J). In addition, we also detected non-specific histological changes in the efferent ductules and vas deferens, which showed an increased duct diameter, thickening duct wall with age (Supplementary Figure S1).

### Region-specific Transcriptome Landscape of the Male Reproductive Tract

**FIGURE 2:**
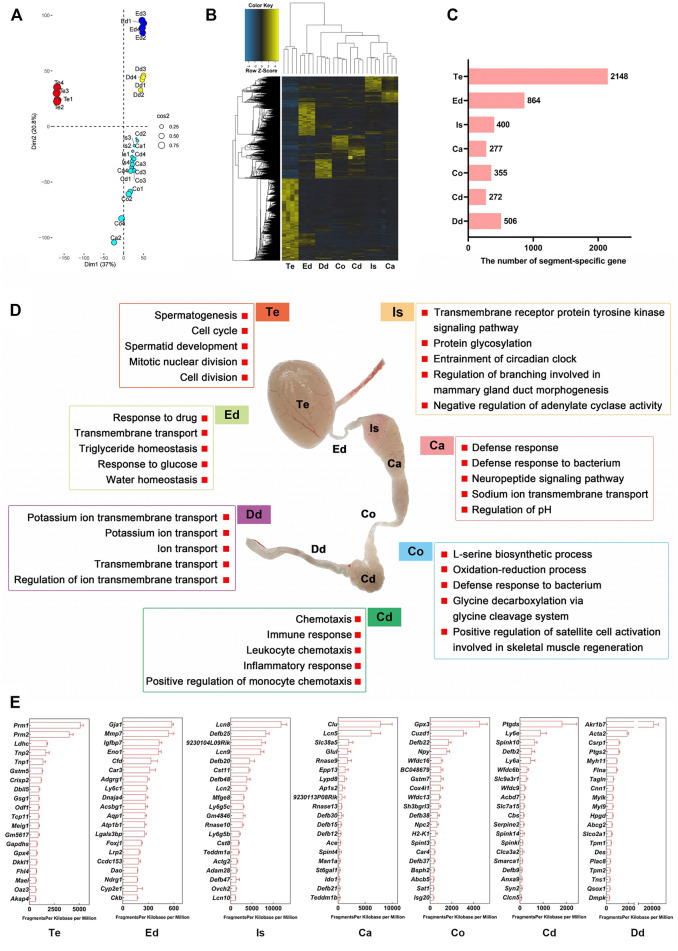
Transcriptome landscape of the male reproductive tract in adult mice. **(A)** Principal component analysis (PCA) of RNA-seq data for seven regions from 3-month-old mice (*n* = 4). **(B)** Heatmap of region-specific genes. Genes with fold change ≥4 relative to levels in the other 6 regions and *p* < 0.05 are defined as region-specific genes. **(C)** Number of region-specific genes. **(D)** Gene Ontology enrichment analysis of region-specific genes. **(E**) Top 20 region-specific genes. Te: testis; Ed: efferent ductules; Is: initial segment; Ca: caput; Co: corpus; Cd: cauda; Dd: ductus deferens.

We performed transcriptome profiling of seven well-defined anatomical regions across the male reproductive tract of 3-month-old adult mice (Figure 2). Genes with fold change larger than or equal to 4 relative to the expression levels in the other 6 regions and *p* < 0.05 were defined as region-specific genes. A principal component analysis (PCA) of the RNA-seq data is showed in Figure 2A and Supplementary Table S2. Interestingly, the unsupervised hierarchical classification, based on region-specific genes, showed a relative affinity between the transcriptome profiles of the initial segment and the caput, corpus, and cauda, and between the vas deferens and epididymal regions. The transcriptome features of the efferent ductules were more similar to those of other post-testicular tube-like structures than to those of the testis (Figure 2B; Supplementary Table S3). These results were consistent with the anatomical distribution of the seven regions across the male reproductive tract. The embryonic origin of the male reproductive tract may explain this coincidence, since the tubular structures of male reproductive duct such as the vas deferens and epididymis are well-known to be derived from the mesonephric duct (also known as Wolffian duct), whereas the testis derived from the primordial genital ridge(Barsoum and Yao, 2006).

We identified a series of region-specific genes of male mouse reproductive tract (Figures 2B,C), which were in broad agreement with those of previous RNA-seq and microarray analyses of murine transcriptome profiles, such as ADAM metallopeptidase domain 28 (*Adam28*) in initial segment, Transmembrane Epididymal Protein 1 (*Teddm1b*) in caput, and serine peptidase inhibitor, Kunitz type 3 (*Spint3*) in corpus epididymis (Johnston et al., 2005; Shi et al., 2021) (reviewed in Supplementary Table S4). GO analysis revealed the functional characteristics of the region-specific genes (Figure 2D; Supplementary Table S5). For example, in the biological process (BP) category, genes expressed in the testis were enriched for the terms “spermatogenesis” and “spermatid development,” genes in the efferent ductules were enriched for the terms “transmembrane transport” and “water homeostasis,” and genes in the caput epididymidis were enriched for “defense response.” The top 20 genes specifically expressed in each region are shown in Figure 2E; Supplementary Table S6. Overall, our bulk RNA-seq analysis provides reliable datasets and shows the transcriptome landscape of the male reproductive tract at an anatomical resolution.

### Verification of Testis- and Epididymis-specific Genes

**FIGURE 3:**
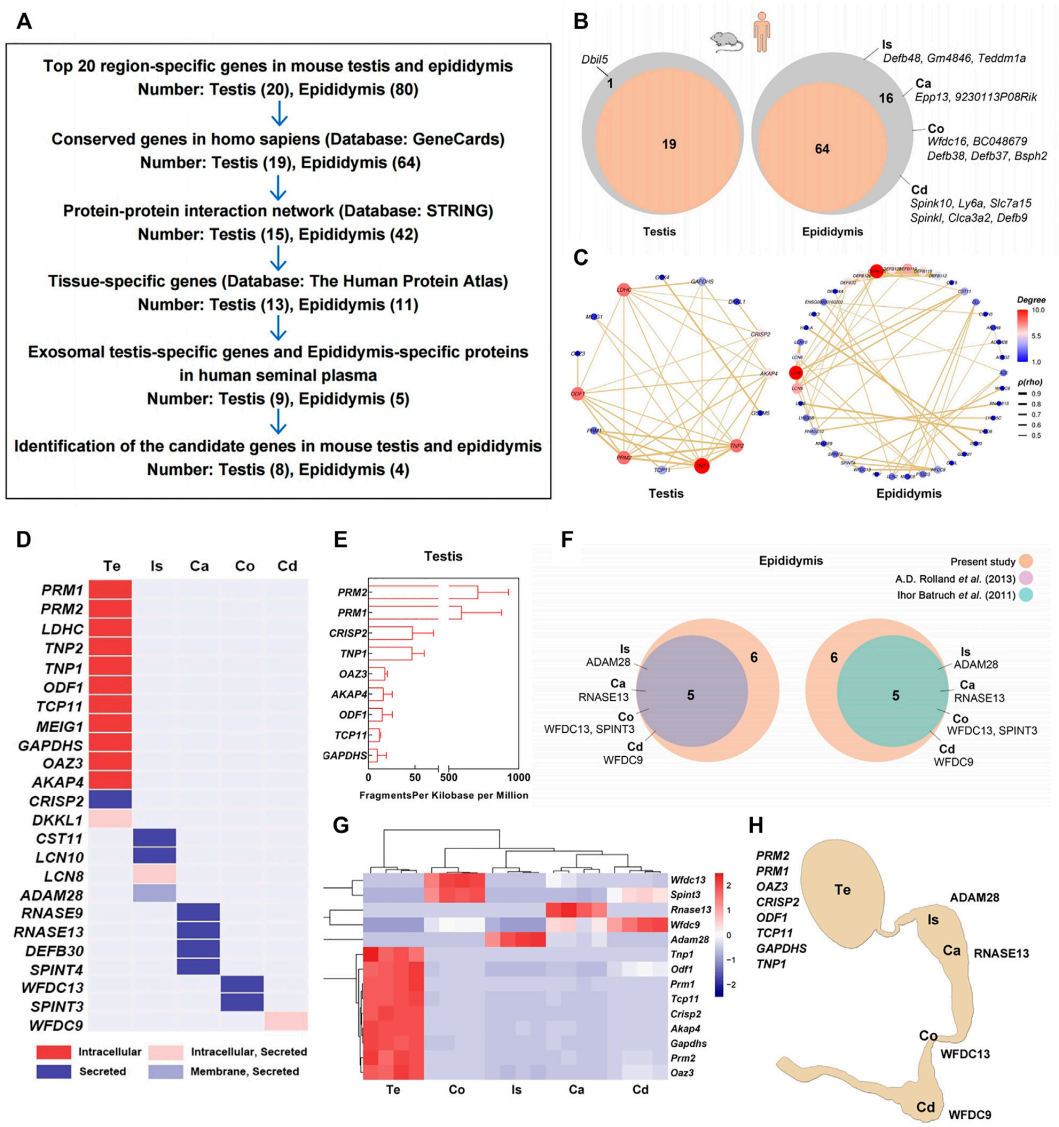
Screening and validation of region-specific markers for the testis and epididymis. **(A)** Workflow diagram of the screening process. **(B)** In total, 19 testis-specific and 64 epididymal region-specific genes were filtered from 100 region-specific genes ranked in the top 20 in these five regions with conserved orthologs in *Homo sapiens* by searches against the GeneCards database. **(C)** In total, 15 and 42 genes plotted in the protein-protein interaction (PPI) network were further picked out using STRING database; **(D)** 13 and 11 out of these 57 genes are confirmed with strict testis- or epididymal tissue-specific expression patterns across human organs in the human protein atlas database. **(E)** Seminal plasma exosomes RNA-seq shows the expression of nine testis-specific genes mRNA in human semen samples (*n* = 3). **(F)** Five epididymal region-specific genes that their products identified in two previously reported proteomics of human seminal plasma. **(G)** Expression patterns of 14 selected region-specific genes according to RNA-seq. **(H)** Epitome of the 12 region-specific genes as candidate markers for the testis and four regions of epididymis. Te: testis; Is: initial segment; Ca: caput; Co: corpus; Cd: cauda.

As region-specific mRNA or protein detection in the seminal plasma enables the noninvasive diagnosis of azoospermia (Li et al., 2012; Xie et al., 2020), we verified the region-specific genes expressed in the mouse testis and four regions of the epididymis, as well their potential uses as seminal markers for the male reproductive tract in humans (Figure 3A). First, we filtered 19 testis-specific and 64 epididymal region-specific genes out of 100 region-specific genes ranked top 20 in these five regions that had conserved orthologs in *Homo sapiens* by browsing the GeneCards database (Figure 3B). Then, 15 and 42 genes plotted in the protein-protein interaction (PPI) network were further picked out using STRING database (Figure 3C). Subsequently, we confirmed 13 and 11 out of these genes with strict testis- or epididymis-specific expression pattern across the human organs by Human protein atlas database (Figure 3D). Most of the products of these 13 testis-specific genes were germ cell-specific and non-secretory proteins, while all 11 epididymis-specific gene products were secretory proteins likely to be detected in human seminal plasma (Figure 3D). Therefore, we further sequenced the human seminal exosomal RNA profiles, compared seminal RNA-seq data with these 13 testis-specific genes, and obtained nine testis-specific genes present in human seminal plasma (Figure 3E; Supplementary Table S7). Additionally, we compared 11 epididymis-specific genes with two prior proteomes of human seminal plasma (Batruch et al., 2011; Rolland et al., 2013), yielding five candidate genes detected in both proteomic datasets (Figure 3F; Supplementary Table S7). According to our RNA-seq data, these 14 region-specific genes in mice showed typical region-specific expression patterns (Figure 3G) and had invariant expression levels in 3-month-old and 21-month-old mice (Supplementary Figure S2).

We validated the expression patterns of these 14 region-specific genes in the testis and epididymis by qPCR (Supplementary Figure S3). Except for Transition protein 1 (*Tnp1*) and *Spint3*, the mRNA expression levels of the other 12 genes (eight for the testis and four for epididymal regions) were significantly enriched in the corresponding regions of the mouse testis and epididymis. The expression of Protamine 2 (*PRM2*) in the human testis was also validated by immunofluorescence staining, showing exclusive expression within the seminiferous tubules and sperm (Supplementary Figure S4). Collectively, we screened and validated 12 region-specific genes that are potential markers for the testis and the four regions of the epididymis (Figure 3H). This finding was a windfall benefit of this transcriptomics analysis, meanwhile, it also verified the reliability of our sequencing data.

### Age-Related Transcriptional Changes in the Male Reproductive Tract

**FIGURE 4:**
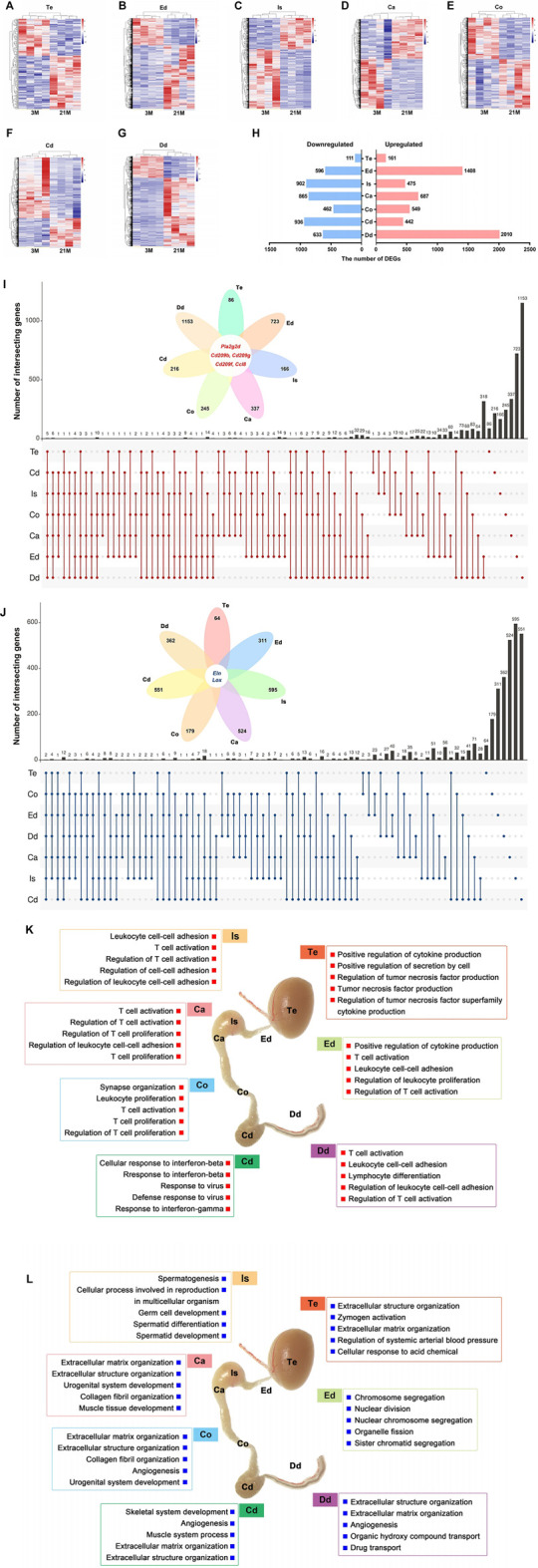
Age-related transcriptional changes in the male reproductive tract. **(A–G)** Heatmaps of age-related differentially expressed genes (DEGs) in seven regions from 3-month-old (*n* = 4) and 21-month-old (*n* = 4) mice. Genes with fold change ≥1.5 (upregulated or downregulated) and *p* < 0.05 were defined as age-related DEGs. **(H)** Numbers of age-related DEGs. **(I)** Upset plot shows the intersection of the upregulated DEGs and **(J)** downregulated DEGs. **(K)** Gene Ontology (GO) enrichment analysis of the upregulated DEGs and **(L)** downregulated DEGs. Te: testis; Ed: efferent ductules; Is: initial segment; Ca: caput; Co: corpus; Cd: cauda; Dd: ductus deferens.

We set out to characterize age-related transcriptome changes in each individual region of male reproductive tract by comparing the datasets from the 3-months and 21-months mice. The genes with at least 1.5-foldchange (upregulated or downregulated by FC ≥ 1.5) were defined as age-related differentially expressed genes (DEGs). The heatmaps of age-related differentially expressed genes (DEGs) showed intra-group clustering at the transcript level (Figures 4A–G). Numbers of age-related DEGs also showed region-dependent patterns (Figure 4H; Supplementary Table S8). Surprisingly, the testis showed a limited number of age-related DEGs (Figures 4A,H). There were 2926 upregulated and 2586 downregulated age-related DEGs in all seven regions of the male reproductive tract (Figures 4I,J). We also assessed overlap in gene expression changes among all seven regions. Five genes (*Pla2g2d*, Cd209 antigen b, Cd209 antigen f, Cd209 antigen g, and C-C motif chemokine ligand 8) were commonly upregulated in all seven regions, while two genes (Elastin and Lysyl oxidase) were commonly downregulated at 21 months (Figures 4I,J; Supplementary Table S9).

To interpret age-related functional changes, we analyzed the biological processes involved with these age-related DEGs. Of note, GO enrichment analysis revealed that the upregulated DEGs were largely associated with BP terms related to the immune response (Figure 4K; Supplementary Table S10), such as “Positive regulation of cytokine production” in testis, “T cell activation” and “leukocyte cell-cell adhesion” in post-testicular regions. Downregulated DEGs were mainly enriched for “Extracellular matrix organization,” “Angiogenesis,” and “Collagen fiber organization” (Figure 4L; Supplementary Table S9), suggesting that the structures of reproductive organs may undergo degenerative changes. In general, the functional analysis revealed increased immune response activities in the aging male reproductive tract.

### Pla2g2d Is a Candidate Biomarker for Testis and Epididymis Aging

**FIGURE 5:**
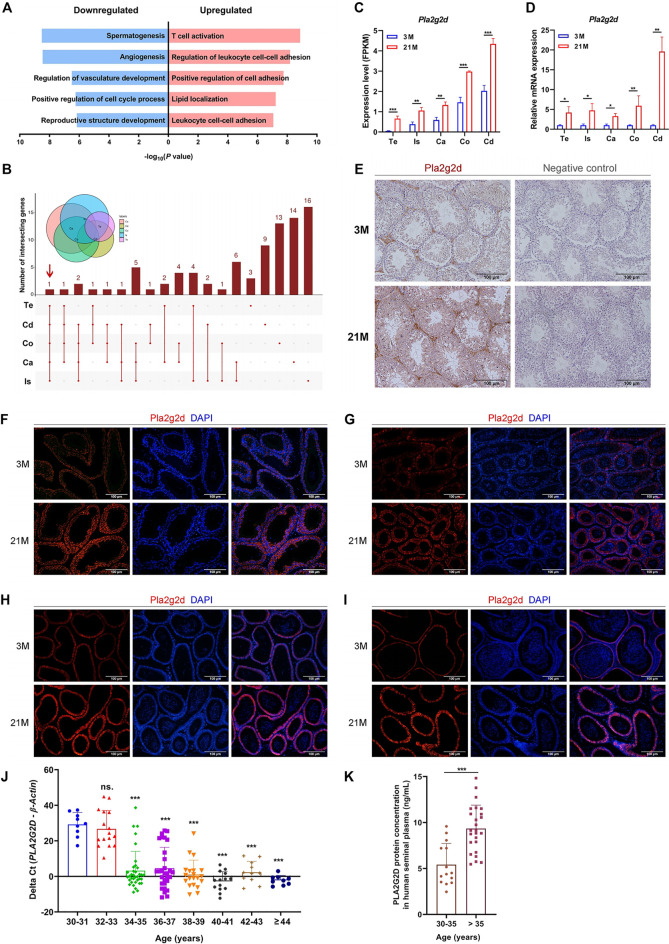
Pla2g2d as a candidate biomarker for male reproductive aging. **(A)** Gene Ontology enrichment analysis of age-related DEGs in the testis and epididymis. **(B)** UpSet plot of the intersection of upregulated DEGs related to “T cell activation.” **(C)** Expression patterns of *Pla2g2d* in the testis and four regions of the epididymis from RNA-seq data and validated by **(D)** qPCR in 3-month-old (*n* = 6) and 21-month-old (*n* = 6) mice. **(E–I)** The expression of Pla2g2d increased with age, as determined by immunohistochemistry in mouse **(E)** testis and by immunofluorescence staining in **(F)** the initial segment, **(G)** caput, **(H)** corpus, and **(I)** cauda. **(J)** *PLA2G2D* mRNA levels in human seminal plasma samples (*n* = 145) at different ages validated by qPCR. *PLA2G2D* mRNA levels were evaluated by the delta Cycle threshold (*Pla2g2d* minus β-actin). **(k)** PLA2G2D protein expression in human seminal plasma samples (*n* = 40) validated by ELISA. *p < 0.05, **p < 0.01, ***p < 0.001; ns.: no significance; Te: testis; Is: initial segment; Ca: caput; Co: corpus; Cd: cauda; 3 M: 3-months; 21 M: 21-months.

Since the testis and epididymis are two of the most important organs for reproductive function, we conducted GO enrichment analysis of age-related DEGs in the testis and epididymis. The upregulated DEGs enriched for functions related to “T cell activation” (Figure 5A). According to the UpSet plot (Figure 4I), *Pla2g2d* was the only common upregulated DEG related to “T cell activation” in all five regions (Figure 5B; Supplementary Table S11). The expression patterns of *Pla2g2d* with age in the testis and four regions of the epididymis in mice were validated by qPCR (Figures 5C,D). And due to the interference of testicular autofluorescence, immunofluorescence staining of Pla2g2d could not be done in the testis. Therefore, we carried out immunohistochemistry (IHC) for Pla2g2d in the mouse testis, which confirmed the upregulated expression level with age (Figure 5E). The immunofluorescence staining of Pla2g2d in the four regions of mouse epididymis shows the increasing protein expression trend of Pla2g2d (Figures 5F–I), which is consistent with our RNA-seq data.

To determine whether human PLA2G2D is also upregulated in seminal plasma with age, we collected 145 human semen samples from consecutive outpatients for fertility examination from 30 to 50 years old with normal semen parameters, which all met the standard of the fifth edition of the WHO manual of semen test. As validated by qPCR and ELISA, PLA2G2D expression at both the mRNA and protein levels in human semen plasma remarkably increased with age (Figures 5J,K). It is worth noting that the cut point of the elevated expression level of PLA2G2D appears at the age of 35 years (*p* < 0.05) (Figures 5J,K). The level of *PLA2G2D* mRNA is almost undetectable until the age of 35, and surges since then (Figure 5J), similar result was also demonstrated at the protein level (Figure 5K). These results suggest that Pla2g2d plays a critical role in the aging of the testis and epididymis and could be a promising biomarker of male reproductive aging in humans.

### Characteristics of Epididymal Aging

**FIGURE 6:**
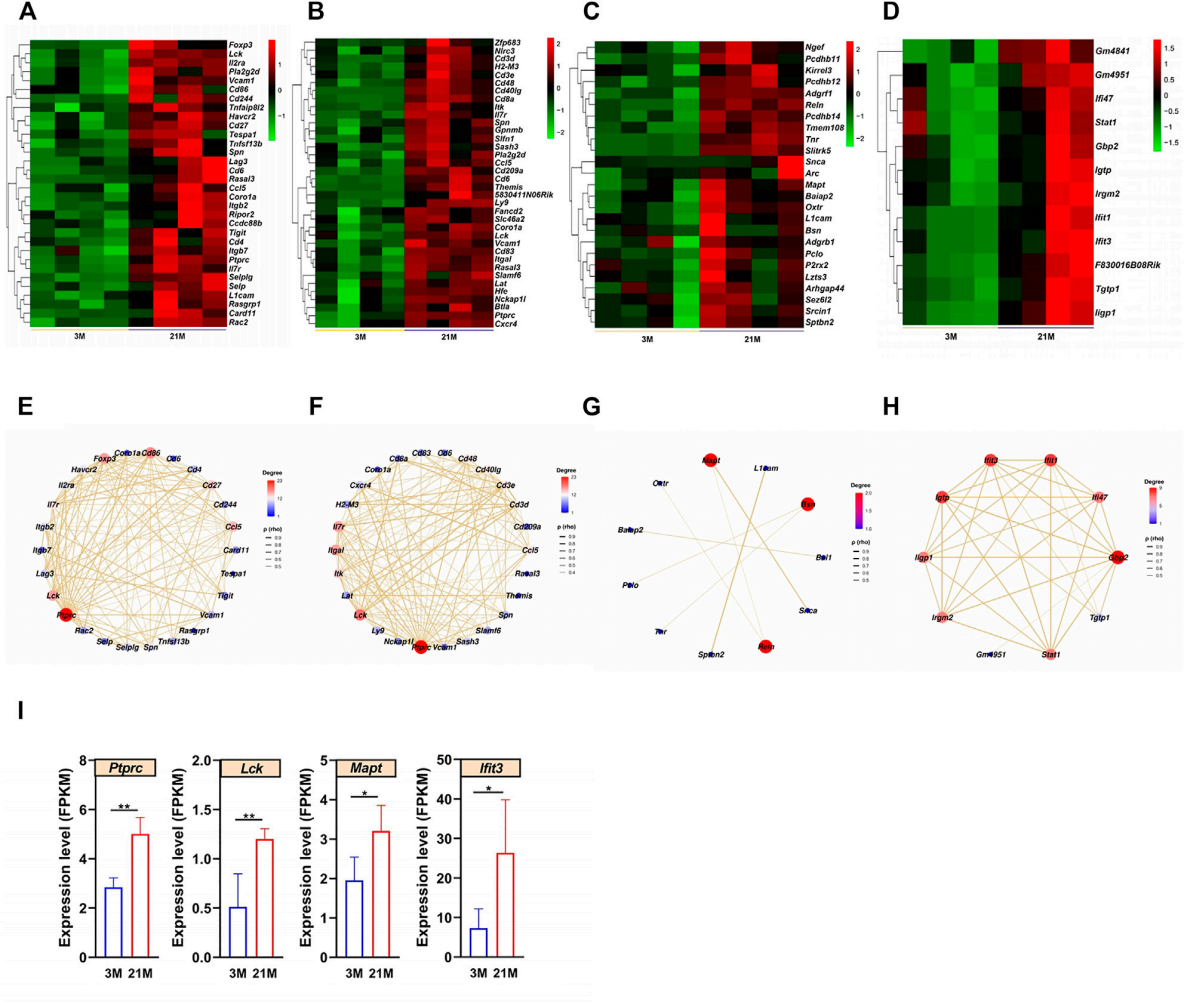
Analysis of aging characteristics in the epididymis. **(A–D)** Heatmaps of upregulated differentially expressed genes (DEGs) enriched to the most significant Gene Ontology (GO) terms in the **(A)** initial segment, **(B)** caput, **(C)** corpus, and **(D)** cauda. **(E–H**) Protein–protein interaction (PPI) networks based on the upregulated DEGs and significant GO terms in four regions of the epididymis. **(I)** Expression levels of *Ptprc*, *Mapt*, *Lck*, and I*fit3* increased with age according to the qPCR (*n* = 6). 3 M: 3-months; 21 M: 21-months.

Previous histological analyses showed that epididymal aging occurs in a region-dependent manner (Figure 1J); accordingly, we focused on aging in the epididymis for a detailed analysis. The upregulated DEGs related to the top biological processes with the maximal -log_10_ (adjust *P*-value) in each region of the epididymis were analyzed (Figures 6A–D; Supplementary Figure S5). The STRING database was further used to construct Protein–protein interaction (PPI) networks of the upregulated DEGs involved in these top biological processes in each region of the epididymis (Figures 6E–H). By qPCR, we validated the expression patterns of several hub genes with high degree values in the PPI network, including Protein tyrosine phosphatase receptor type C (*Ptprc*) in the initial segment, Lymphocyte protein tyrosine kinase (*Lck*) in the caput epididymidis, Microtubule associated protein tau (*Mapt*) in the caput epididymidis, and Interferon induced protein with tetratricopeptide repeats 3 (*Ifit3*) in the cauda epididymidis. All four genes exhibited increased expression with age (*p* < 0.05) (Figure 6I), in accordance with the RNA-seq data.

## Discussion

In this study, we mapped the general transcriptome of the male mouse reproductive tract from both temporal and spatial perspectives using bulk RNA-seq data. Specifically, we identified a series of region-specific genes with potential diagnostic value for male infertility and characterized age-related transcriptomic changes. Further analyses showed that increased immune response activity is associated with male reproductive tract aging, and identified *Pla2g2d*, an immune-associated gene, as a candidate biomarker for male reproductive aging.

Unlike the relatively abrupt decline that occurs in women at the time of menopause (Broekmans et al., 2009; Wang et al., 2020), the change in men is gradual during aging. Indeed, it has some adverse consequences in reproductive ability and testicular endocrine disruption. In males, seminal parameters begin to decline at approximately 35 years of age (Stone et al., 2013). Similarly, peak values for semen parameters have been detected at 30–35 years of age (Levitas et al., 2007). Moreover, male reproductive endocrine declines with increasing age and may start as soon as 30 years of age, demonstrated by decreases in serum testosterone, bone mineral density, and muscle strength (Wu et al., 2008; Salonia et al., 2019; Snyder, 2019). Specifically, epidemiological studies have demonstrated a 0.5–1.5% per year decrease in circulating total testosterone concentrations and a 2–3% per year decrease in free testosterone concentrations in men beyond the age of 30 years (Salonia et al., 2019). Moreover, hypogonadism is a common disorder in aging men with a significant percentage of men over 60 years of age (Surampudi et al., 2012). Male mice over 18 months are generally recognized as the aged ones with remarkable reproductive aging, which have been characterized in previous studies (Li et al., 2017, 2020). However, according to a recent multi-omics analysis on aging in human and mice, the 18 months old male mice were only equal to human aged 56 years, while the 21 months old male mice were equal to human aged 63 years (Schaum et al., 2020). Therefore, we selected male mice aged 21 months rather than 18 months to ensure the comparability with human. Our phenotypic analysis suggested a disruption in reproductive function in 21-month-old mice. In addition, the loss of muscle strength, reduced bone density, and increased fat mass are also thought to be associated with the age-related testosterone deficiency, or hypogonadism (Zirkin and Tenover, 2012), as observed in the current study. Collectively, our findings support the significance of aging-related changes in the male reproductive tract of mice.

The impact of aging on male gonads (testes) remains to be clarified. Here, we found that aging testes exhibit disruptions in spermatogenesis and testosterone synthesis as well as abnormal histological changes in mice. However, β-Gal-positive cells were only found in the interstitia of aging testes, suggesting that cells residing in the testicular interstitial space, such as Leydig cell and macrophages, are crucial to the age-related microenvironment of the testis (Zhang et al., 2020a; Wang et al., 2021). As previously reported, the reduced expression levels of testosterone synthetic enzymes (cytochrome P450 family 11 subfamily A member 1 (*Cyp11a1*), 3 Beta-Hydroxysteroid Dehydrogenase (*3βhsd*), Cyp17a1, and 17βhsd) (Midzak et al., 2009), insulin like 3 (*Insl3*) (Paust et al., 2002), and several antioxidant family genes (Cu/Zn Superoxide Dismutase, and glutathione peroxidase 1) (Cao et al., 2004) were thought to be associated with Leydig cell senescence. However, in our transcriptomics analysis, only minor differences were found between young adult and elderly testicular transcriptomes in mice. Except for *Cyp11a1* and *3βhsd6*, there were no changes in the expression of genes previously identified as potential Leydig cell senescence markers (*e.g.*, *Insl3*). It is possible that the senescence of testicular cells is not synchronized, so that transcriptomic contents from all complex cell types of the testis may dilute the age-related changes in senescent cells. Thus, to gain a deeper molecular understanding of aging of the testis, a single-cell level RNA-seq by enriching for somatic cells in the testicular interstitial space are still needed (Green et al., 2018).

Given the complex anatomy of the epididymis, epididymal aging occurs in a region-dependent manner, especially in the proximal region. As shown by our anatomical observation, the blood supply is richest in the proximal epididymis, similar to a previous finding (Markey and Meyer, 1992). It has also been argued that caput epididymal mouse sperm were capable of supporting full embryonic development (Zhou et al., 2019). These lines of evidence suggest an important role of the proximal region of the epididymis during epididymal transit of sperm (Zhou et al., 2019). Therefore, we believe that the initial segment and caput epididymidis could be two of the most interesting regions for further detailed studies.

Several studies have focused on the changes of gene expression in epididymis with age. Epididymal function-related genes, including 5α-reductase type 1 and 2 (*Srd5a1/Srd5a2*) and proenkephalin (*Penk*), could be affected by aging in a region-specific manner (Viger and Robaire, 1995). Moreover, antioxidant enzyme activity in the epididymis may also decrease with age (Jervis and Robaire, 2002; Zubkova and Robaire, 2004). In our analysis of the murine epididymis, *Srd5a1* and *Srd5a2* showed no age-related changes, whereas *Penk* was downregulated by 0.47-fold in the caput epididymidis of mice. Additionally, we identified several key factors involved in epididymal aging of different regions. Of note, *Ptprc* is related to the differentiation of memory T cells and is related to the aging of the brain and optic nerve (El-Sayyad et al., 2014; Lagou et al., 2018; Mukherjee et al., 2019), whereas *Lck* is associated with renal aging (Larbi et al., 2004; Kim et al., 2019). Moreover, *Mapt* is a well-known gene encoding the microtubule-associated protein tau and has been implicated in Parkinson’s disease (Dhib-Jalbut, 2015). And *Ifit1* is related to brain aging and neuromuscular aging (Dhib-Jalbut, 2015; Zhang et al., 2020b). These genes may also play critical roles in the aging of each region of the epididymis.

We found that increased immune responses are generally associated with male reproductive tract aging. The male reproductive tract usually has immune privilege due to the blood–testis and blood–epididymis barriers. However, this balance may be disrupted during aging. With advancing age, a mild chronic proinflammatory state is commonly observed in the male reproductive tract (Frungieri et al., 2018). Inflammation in the male reproductive tract can be induced by infectious agents, urine reflux, hormonal changes, or other noninfectious factors (Azenabor et al., 2015). In the epididymis, it was reported that helper T lymphocytes, cytotoxic T lymphocytes, and monocytes increased within the epithelium of aged rats in a region-specific manner (Serre and Robaire, 1999). By contrary, genes related to “collagen fibril organization” and “extracellular matrix organization” were downregulated with age, including several fibrillar collagen family genes (*Col1*, *3*, *4*, and *5*) and Elastin (*Eln*, encoding elastic fibers). *Eln* expression was also downregulated in all seven regions of the male reproductive tract. These results indicate that the aging of the epididymis and other duct-like structures is closely associated with extracellular matrix changes (Birch, 2018; Romero-Ortuno et al., 2020). Considering that the expression profiles of the male reproductive tract can be affected by testosterone and other sex hormones (Hu et al., 2014; Ramli et al., 2019), the potential for some of these transcriptome changes may be secondary to the decline in testicular endocrine function cannot be ruled out. Several members of Defensin Beta family (*Defb* 18, 19, 20, 39, 40) were both age-related and regulated by orchiectomy and testosterone replacement in mouse caput epididymidis (Hu et al., 2014). In addition, lumicrine signaling from testis also affected the development and function of epididymis (Kiyozumi et al., 2020), and some unknown testicular factors could affect the expression of immunoregulatory genes in the epididymal caput (Wijayarathna et al., 2021). Thus, it is possible that lumicrine factors might also affect epididymis aging.

Further, our dataset provides an RNA-seq resource for identification of region-specific genes and age-related genes which could be used as potential biomarkers. The seminal plasma harbors cell-free DNA, RNA (including miRNA, piRNA, tsRNA, lncRNA, and mRNA), peptides, and proteins released or secreted by different regions of the male reproductive tract. According to our previous study, testis-specific RNA could be used as a predictor of the sperm retrieval rate of patients with non-obstructive azoospermia (NOA) (Xie et al., 2020; Chen et al., 2021). Additionally, germ cell-specific (DEAD-box helicase 4), seminal vesicle-specific (Semenogelin 1), and prostate-specific (Transglutaminase 4) mRNAs in seminal plasma could be markers for identifying the presence of germ cells or the rough localization of complete obstructive azoospermia (Li et al., 2012). Although many studies have explored region-specific genes or proteins, few have evaluated the potential clinical applications of these massive data sets. In this study, we screened for and validated several potential markers, including eight testicular markers and four epididymal markers both for mouse and human. Notably, seminal *PRM1* and *PRM2* were previously identified as biomarkers of assisted reproductive technology outcomes (Sukhikh et al., 2012) or sperm retrieval outcomes in patients with NOA (Haraguchi et al., 2009), while ADAM28 is downstream of Ovochymase 2 in male reproductive lumicrine signaling (Kiyozumi et al., 2020).

We also made our efforts to explore the biomarkers for the male reproductive tract aging in mice. In the present study, *Pla2g2d* was functionally related to the top enriched biological process for the upregulated DEGs in the testis and four regions of the epididymis, as well as one of the common age-related DEGs among all seven regions, suggesting a critical role in male reproductive aging. Moreover, the change in PLA2G2D expression levels in human seminal plasma was detected at approximately 35 years of age, consistent with the reported peak of human semen parameters (Stone et al., 2013) and serum testosterone (Wu et al., 2008; Salonia et al., 2019), suggesting that PLA2G2D is an early indicator of male reproductive decline, preceding decreases in semen parameters. *Pla2g2d* is involved in lung aging, as evidenced by its 50-fold higher expression in middle-aged than in young mice (Vijay et al., 2015). However, very limited literature has focused on the detailed role of Pla2g2d in aging or male reproduction. It will be interesting to investigate *Pla2g2d* and other age-related genes as possible interference targets in male reproductive aging.

In conclusion, this study showed that the dramatic age-related changes occurred in the testis and proximal epididymis, and increased immune response, such as the T cell activation, was closely associated with male reproductive tract aging. Additionally, our findings provide an RNA-seq resource for identification of region-specific genes could be used as potential biomarkers in azoospermia, and suggests that targeting the immune response pathways may be used to delay aging. To gain in-depth understanding of the biology of male reproductive aging, functional experiments of the genes identified in this study are needed. And higher resolution transcriptome studies, such as single-cell RNA sequencing, could be conducted in the testis and proximal epididymis for detailed analysis in the future.

## Data Availability

The datasets presented in this study can be found in online repositories. The names of the repository/repositories and accession number(s) can be found below: GEO repository (GSE181426).
