# Supplementary material for: Anatomical Transcriptome Atlas of the Male Mouse Reproductive System During Aging
Source: Front Cell Dev Biol. 2022 Feb 8;9:782824. doi: 10.3389/fcell.2021.782824 (PMC8861499; doi:10.3389/fcell.2021.782824)

Figure S1. **Representative hematoxylin and eosin staining of the age-related changes of the efferent ductules and vas deferens**. 3M: 3-month; 21M: 21-month.

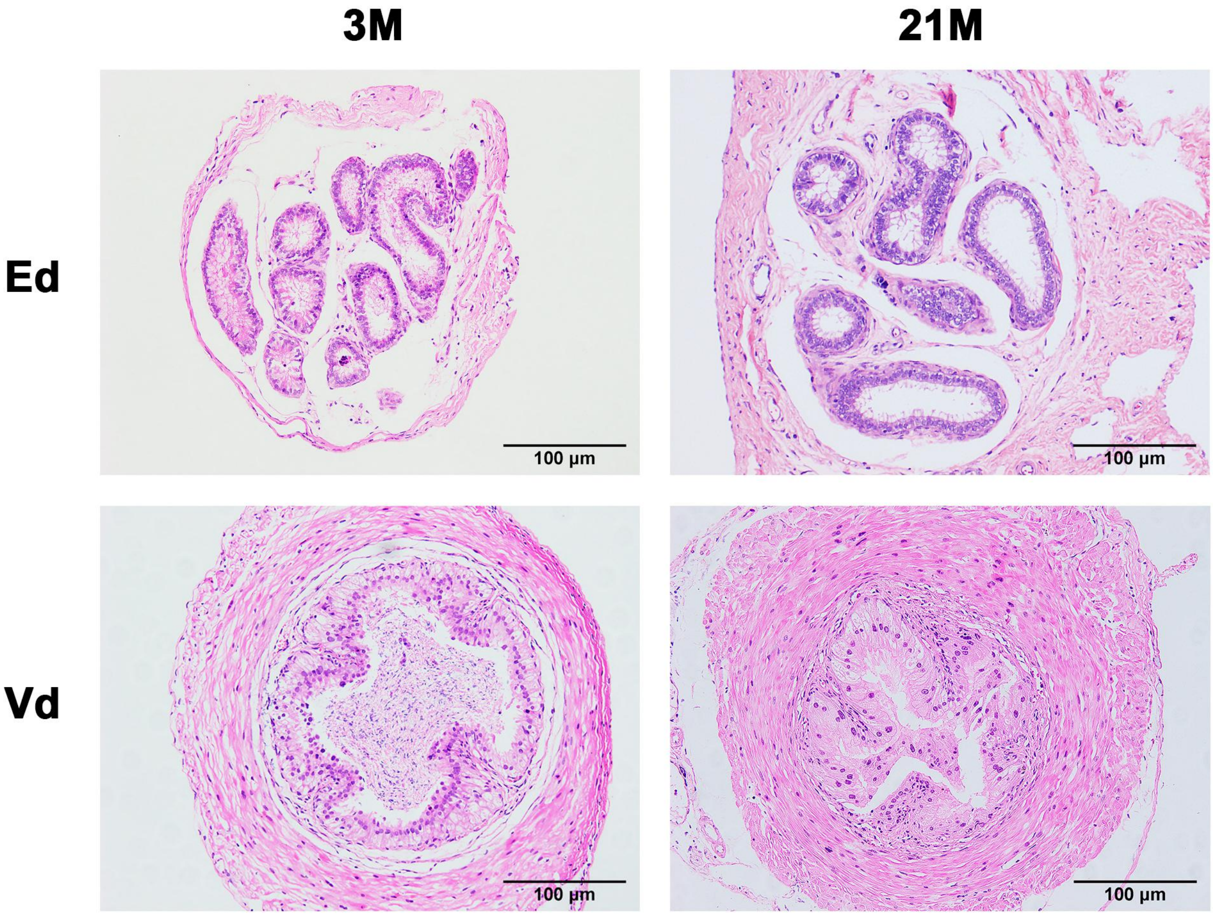

Figure S2. The expression pattern of the 14 selected region-specific genes in aged mice. Prm1, Prm2, Crisp2, Tnp1, Oaz3, Akap4, Odf1, Tcp11, and Gapdhs show region-specific expression in the testis, while Adam28, Rnase13, Spint3, Wfdc13/Wfdc9 show region-specific expression in the four regions of epididymis, respectively, according the RNA-Seq data of the 21-month-old mice (n = 4). Te: testis; Is: initial segment; Ca: caput; Co: corpus; Cd: cauda.

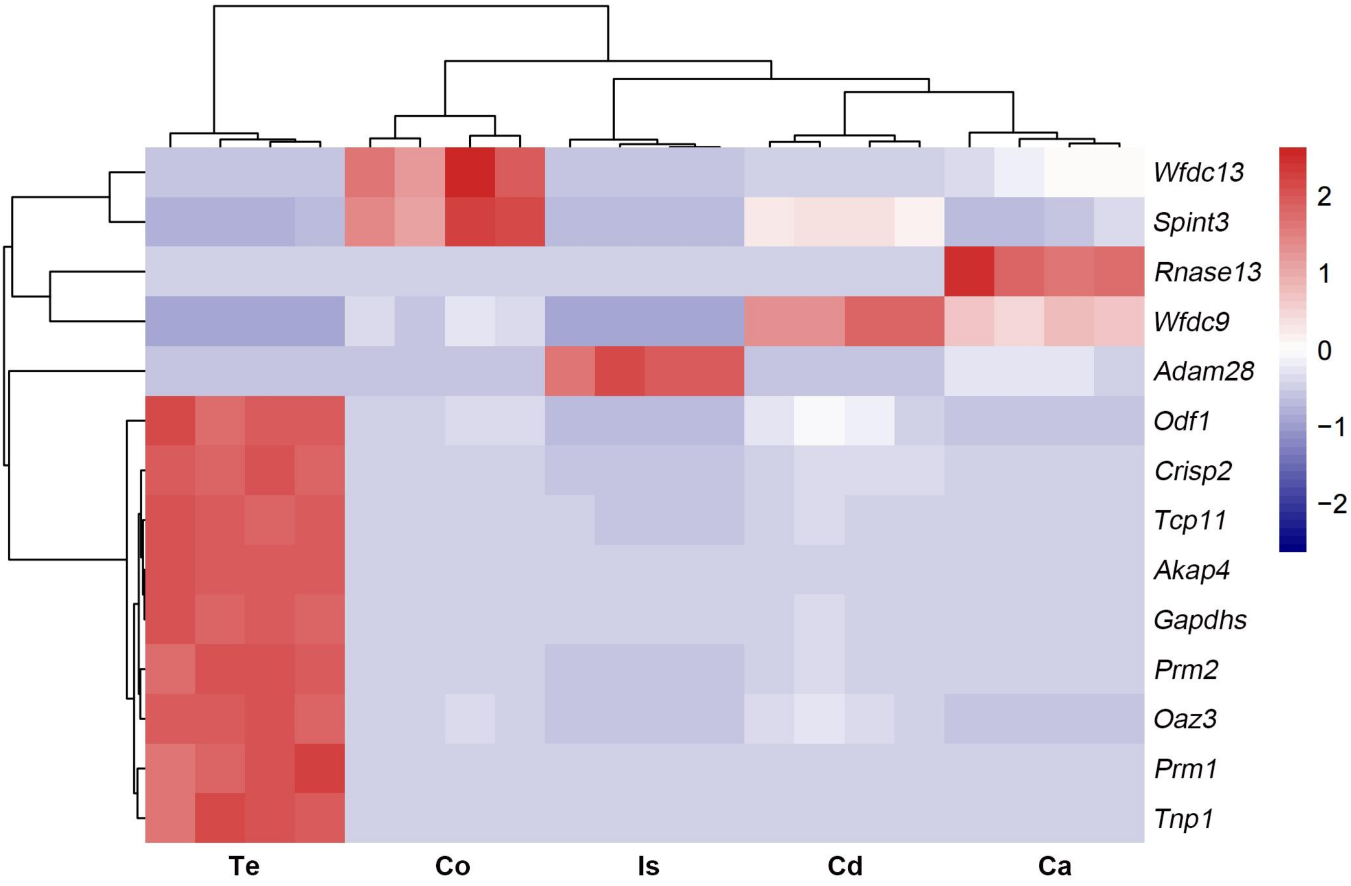

Figure S3. Verification the expression of the 14 selected region-specific genes by quantitative PCR in young mice. As showed by quantitative PCR in 3-month-old mice (n = 4), the Tnp1 and Spint3 expression levels show no significant differences comparing to some other regions of the male reproductive tract. The mRNA expression levels are evaluated by the delta Cycle threshold (delta Ct, Gene minus β-actin) instead of the relative expression, since it is arbitrary to define specific region as baseline for standardization. Te: testis; Is: initial segment; Ca: caput; Co: corpus; Cd: cauda.

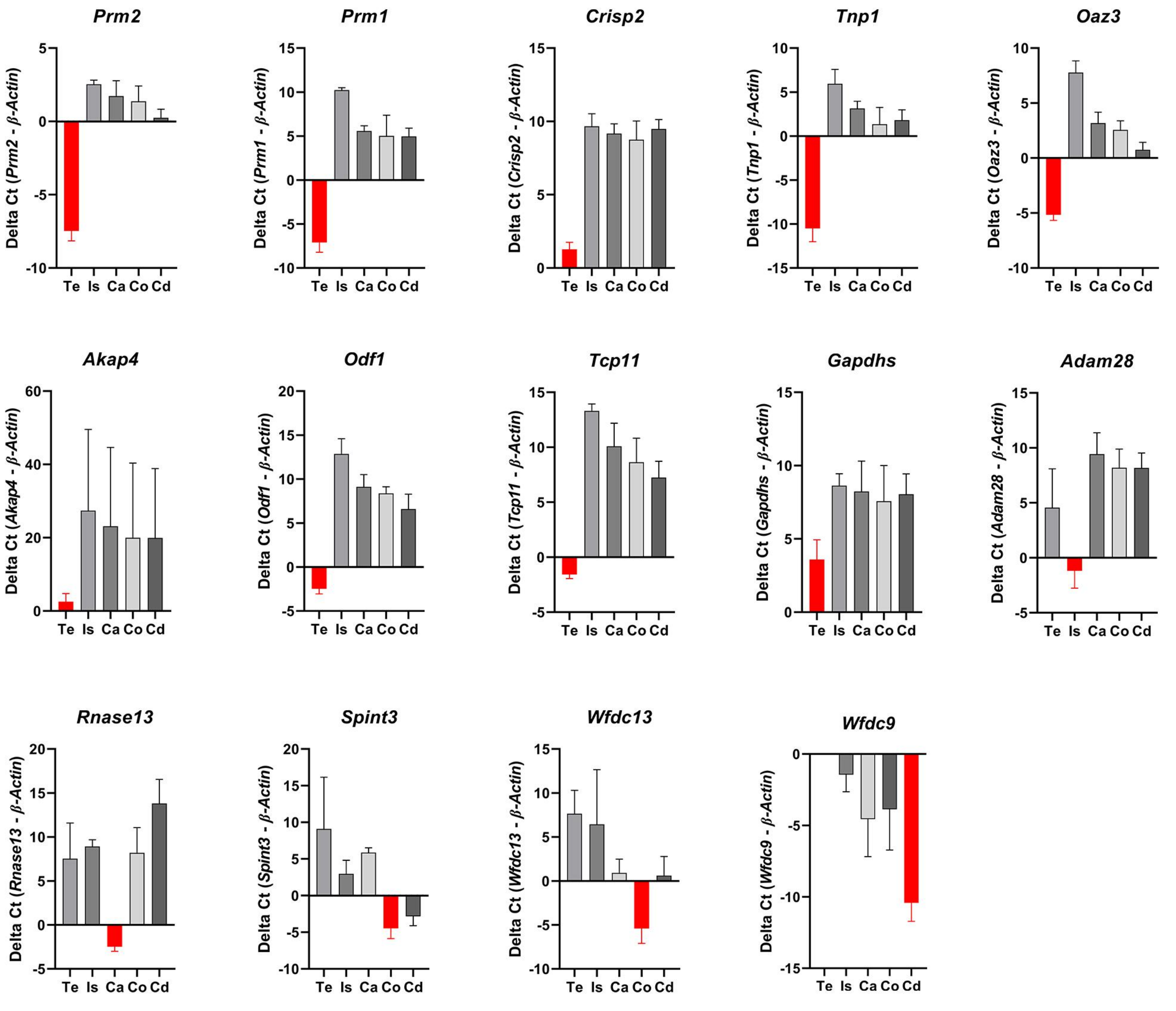

Figure S4. Representative immunofluorescence staining for the PRM2 in the human testis and sperm. The expression of PRM2 shows strict germ cell-specific expression.

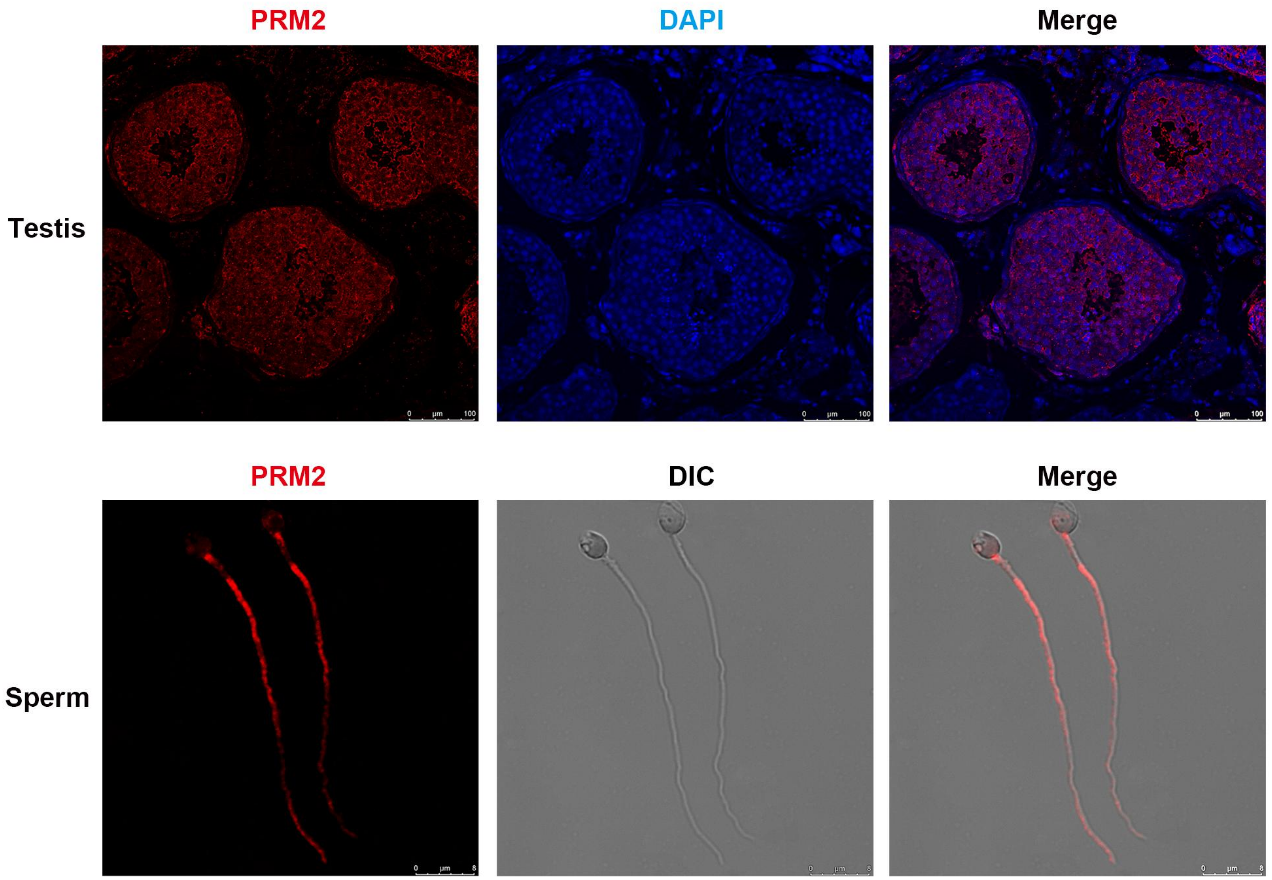

Figure S5. Network plot of the top 5 Gene Ontology terms for the upregulated differentially expressed genes (DEGs) in the epididymis. (A) Initial segment, (B) caput, (C) corpus, and (D) cauda epididymidis.

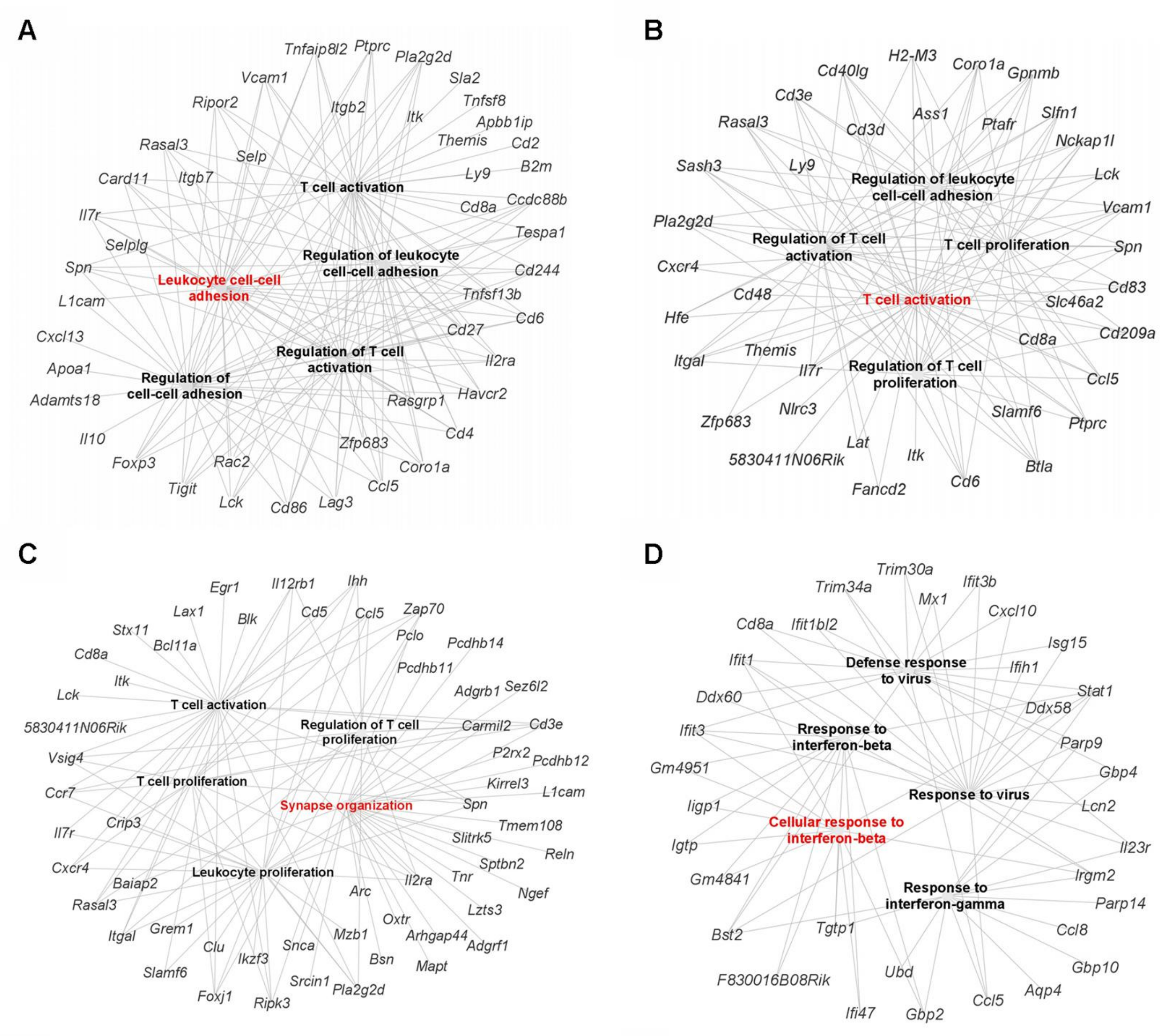

Supplement: Supplementary file 5 [file DataSheet1.PDF]
